# In vitro and in vivo investigation of osteogenic properties of self-contained phosphate-releasing injectable purine-crosslinked chitosan-hydroxyapatite constructs

**DOI:** 10.1038/s41598-020-67886-7

**Published:** 2020-07-14

**Authors:** Kaushar Jahan, Garthiga Manickam, Maryam Tabrizian, Monzur Murshed

**Affiliations:** 1 Faculty of Dentistry, McGill University, 1003 Boulevard Décarie, Montreal, QC H4A 0A9 Canada; 2 Department of Biological and Biomedical Engineering, McGill University, 3775 University Street - Duff Medical Building, Montreal, QC H3A 2B4 Canada; 3 Department of Medicine, McGill University, Montreal, QC Canada; 4 Shriners Hospital for Children, McGill University, Montreal, QC Canada

**Keywords:** Biomaterials - cells, Biomineralization

## Abstract

Bone fracture repair is a multifaceted, coordinated physiological process that requires new bone formation and resorption, eventually returning the fractured bone to its original state. Currently, a variety of different approaches are pursued to accelerate the repair of defective bones, which include the use of 'gold standard' autologous bone grafts. However, such grafts may not be readily available, and procedural complications may result in undesired outcomes. Considering the ease of use and tremendous customization potentials, synthetic materials may become a more suitable alternative of bone grafts. In this study, we examined the osteogenic potential of guanosine 5′-diphosphate-crosslinked chitosan scaffolds with the incorporation of hydroxyapatite, with or without pyrophosphatase activity, both in vitro and in vivo. First, scaffolds embedded with cells were characterized for cell morphology, viability, and attachment. The cell-laden scaffolds were found to significantly enhance proliferation for up to threefold, double alkaline phosphatase activity and osterix expression, and increase calcium phosphate deposits in vitro. Next, chitosan scaffolds were implanted at the fracture site in a mouse model of intramedullary rod-fixed tibial fracture. Our results showed increased callus formation at the fracture site with the scaffold carrying both hydroxyapatite and pyrophosphatase in comparison to the control scaffolds lacking both pyrophosphatase and hydroxyapatite, or pyrophosphatase alone. These results indicate that the pyrophosphatase-hydroxyapatite composite scaffold has a promising capacity to facilitate bone fracture healing.

## Introduction

Current therapies for injury-related bone loss rely on the use of bone grafts taken from the patient (autograft) or a donor (allograft) that require extensive restructuring and involve invasive surgical procedures. These procedures include the bone length and axis corrections with the Ilizarov ring fixator^1^, as well as the debridement of the wound to remove any damaged tissues with the Masquelet technique^2^. Moreover, grafts are associated with risks of infection, donor-site morbidity, and lack of osseointegration to the host bone^3,4^. In order to overcome some of these issues, ceramic-based synthetic bone grafts such as hydroxyapatite (HA) are commonly used in bone repair therapies^5^. HA is a calcium phosphate-based biomineral found in bone and is often used as a bone substitute and delivery system^6^. Cranioplasty performed with porous-HA prostheses, which were designed with inputs from the surgeons, resulted in minimal adverse response and morbidity rates in 51 patients. Also, the lack of mechanical complications and the optimistic clinical data confirmed the high-level safety and good quality performance in these cranial defect repairs^7^. Functional integration of HA macroporous scaffolds implanted in four patients with large bone diaphysis was observed in a 6-year follow-up study for all patients^8^. Further, 66 patients who received a biphasic calcium phosphate substitute, Osteosynt, in various orthopaedic surgeries (femur, hip, ankle, tibia, knee, wrist, humerus and clavicle) showed good to excellent results by clinical analyses (score 0 to 5) of bone formation, range of movement, osseointegration of the bioceramic and healing of the defect^9^.

The surgical insertion of HA bioceramics^7–12^ and the difficulty of retaining them in bone defects create some limitations for their use. This issue can be addressed by making a composite scaffold, preferably injectable, using a polymer as a matrix. Since the early 1990s, various biomaterials have been exploited for the fabrication of ceramic-polymer bone scaffolds^13–16^. Among all the biomaterials, chitosan scaffolds have received significant attention due to their similarity to the extracellular matrix (ECM) present in the mineralized tissues. Chitosan is a linear polysaccharide derived from natural chitin and is composed of an alternating copolymer of (1 → 4)-2-acetamido-2-deoxy-β-d-glucan (N-acetyl-d-glucosamine) and (1 → 4)-2-amino-2-deoxy-β-d-glucan (d-glucosamine) linked by β (1 → 4) linkages. When implanted in vivo, depolymerization of chitosan can take place through the action of enzymes like lysozyme, lipases, and glucosaminidases^17^. Chitosan has excellent biocompatibility, low cytotoxicity, and immune response, and can promote cell adhesion, proliferation, and new tissue formation^18^. Further, it is easy to modify chitosan chemically to generate scaffolds with desired properties. Due to these beneficial features, pH- and temperature-responsive chitosan-based hydrogels have been widely used for tissue engineering applications^19–21^.

Despite all the beneficial properties, chitosan’s relatively weak mechanical properties may fall short for bone repair applications. To address this issue, linear chitosan chains are cross-linked by purine nucleotides, and biominerals HA and β-TCP are added to form biocomposites^22–24^. A purine nucleotide consists of an adenosine or guanosine molecule connected to one, two or three phosphate groups^25^. Purine nucleotides can act as ligands for P2X receptors, which may, in turn, stimulate the differentiation of human mesenchymal stem cells (MSCs) to osteoblasts^26–29^ and promote skeletal tissue development^30,31^. These studies indicate that the use of purine nucleotides to crosslink chitosan may have beneficial effects on bone fracture healing.

While crosslinked chitosan composites may have desired mechanical properties for bone repair applications, their degradation may lead to the generation of inorganic pyrophosphate (PP_i_), a known inhibitor of physiologic ‘hard tissue’ mineralization^32,33^. We demonstrated that the addition of the enzyme pyrophosphatase (PPase) to the scaffold breaks down PP_i_ into two phosphate ions, and as such the scaffold can be modified to act as a reservoir for these ions essential for ECM mineralization in vitro and in vivo. To take advantage of the already available purine crosslinked chitosan, we developed a one-step new formulation of purine crosslinked-chitosan uniformly encapsulating HA and PPase to simulate the bone microenvironment and facilitate bone regeneration^34,35^.

An ideal biomaterial for bone fracture healing should be biodegradable, biocompatible, and should mimic the bone microenvironment with the essential cellular and extracellular cues promoting cell migration, proliferation and differentiation. Ideally, the material should carry interconnected pores of desired sizes (100–200 μm), allowing adequate cell infiltration to form viable pre-osteoblasts-laden scaffolds, diffusion of nutrients and wastes, and eventually resulting in ECM synthesis and its proper mineralization^36^. Based on these criteria, we hypothesized that our injectable chitosan scaffold with encapsulated HA and PPase will be a suitable material for bone tissue regeneration with improved bone fracture healing capacity. We tested this hypothesis by performing a systematic study using regenerative medicine and a combination of a scaffold, cells, and essential factors for minimally invasive bone tissue reconstruction^37^. We studied the behaviour of murine MC3T3 pre-osteoblasts encapsulated in different chitosan scaffolds with various combinations of HA and PPase in vitro and compared their usability in bone fracture repair in a mouse model of tibial fracture fixed with an intramedullary rod.

## Results

### MC3T3-E1 cells proliferate in chitosan-ceramic composite scaffolds

**Figure 1:**
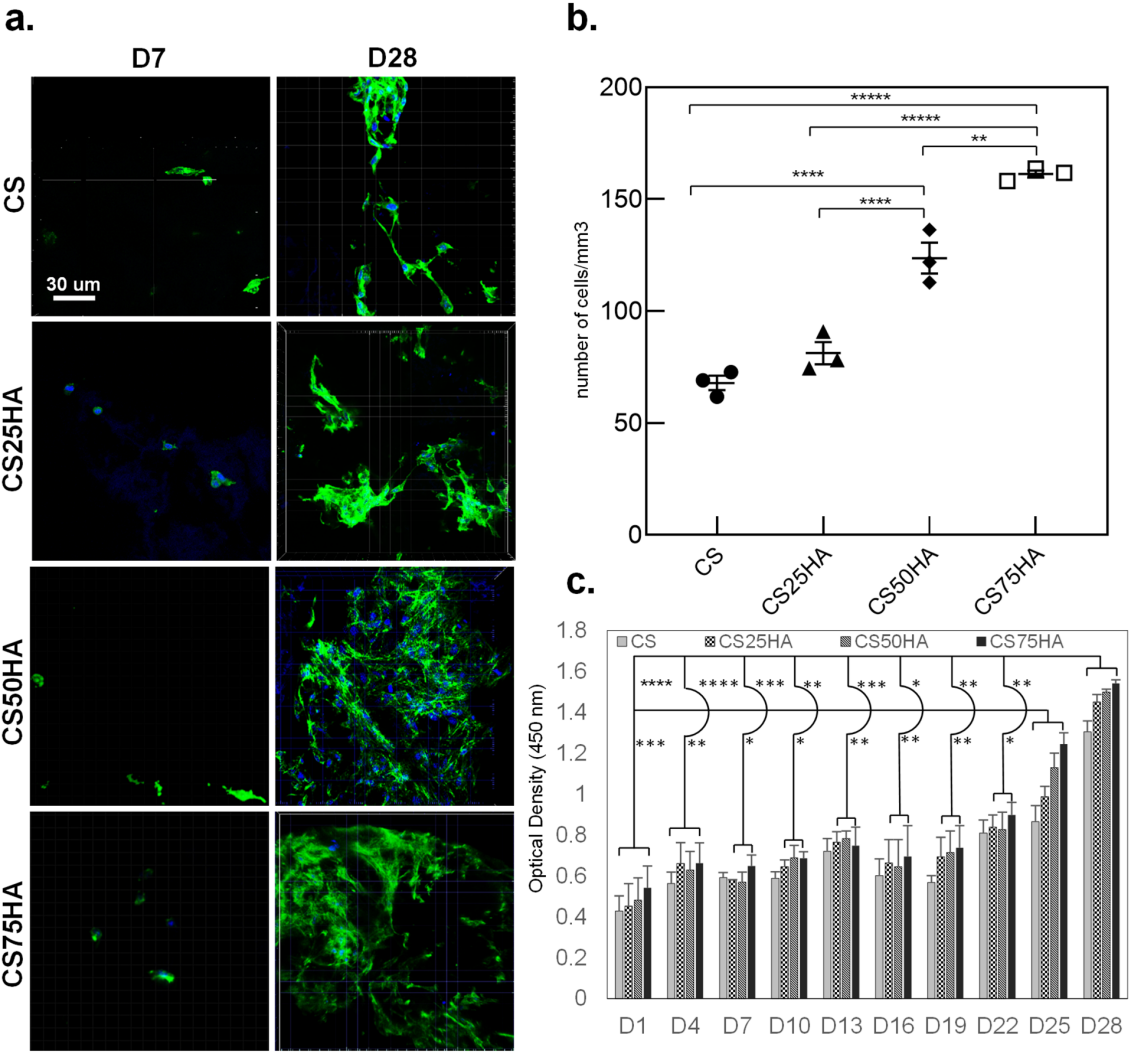
Confocal microscopy observations of MC3T3-E1 cells adhesion at (**a**) days 7 and 28 and (**b**) cell count number at day 28. The actin filaments are stained in green with phalloidin, and the nuclei are stained in blue with DAPI. (**c**) Alamar Blue colorimetric assay showing the metabolic activity of the encapsulated MC3T3-E1over 28 days. Mag.: 40X, scale bar = 30 μm. *p* < 0.0001(****), *p* < 0.001(***), *p* < 0.01(**) and *p* < 0.01(*).

Table 1 presents the different compositions of scaffolds used for MC3T3-E1 cells’ proliferation assay. The scaffolds were cultured in the maintenance medium for 28 days and examined by fluorescent confocal microscopy after staining for actin cytoskeleton and nuclei. A higher number of cells on day 28 in comparison to day 7 in all the scaffolds indicates the suitability of the scaffold for cell proliferation (Fig. 1a). Proliferation was higher on all the scaffolds with HA, the highest being on 75 (wt%) HA composites, after both 7 and 28 days. CS75HA showed over twice the number of cells per mm^3^ than CS (*p* < 0.0001). CS50HA also showed a significantly higher number of cells in comparison to CS on both days (Fig. 1b).

**Table 1:** Concentrations for proliferation, morphology and attachment study.

Group	Abbr	Chitosan (mg/ml)	GDP (mg/ml)	Hydroxyapatite (mg/ml)
Chitosan	CS	6	100	0
Chitosan/HA	CS25HA	6	100	1.5
	CS50HA	6	100	3.0
	CS75HA	6	100	4.5

These results were also confirmed by measuring the metabolic activities of the encapsulated cells in various scaffolds using Alamar Blue assay and monitoring the change in colour of the medium over time by spectrophotometer. Significantly higher metabolic activities of cell encapsulated in CS50HA and CS75HA groups than the control CS group was found at all time points (Fig. 1c).

### SEM and fluorescence microscopy showed cell attachment to the scaffold

**Figure 2:**
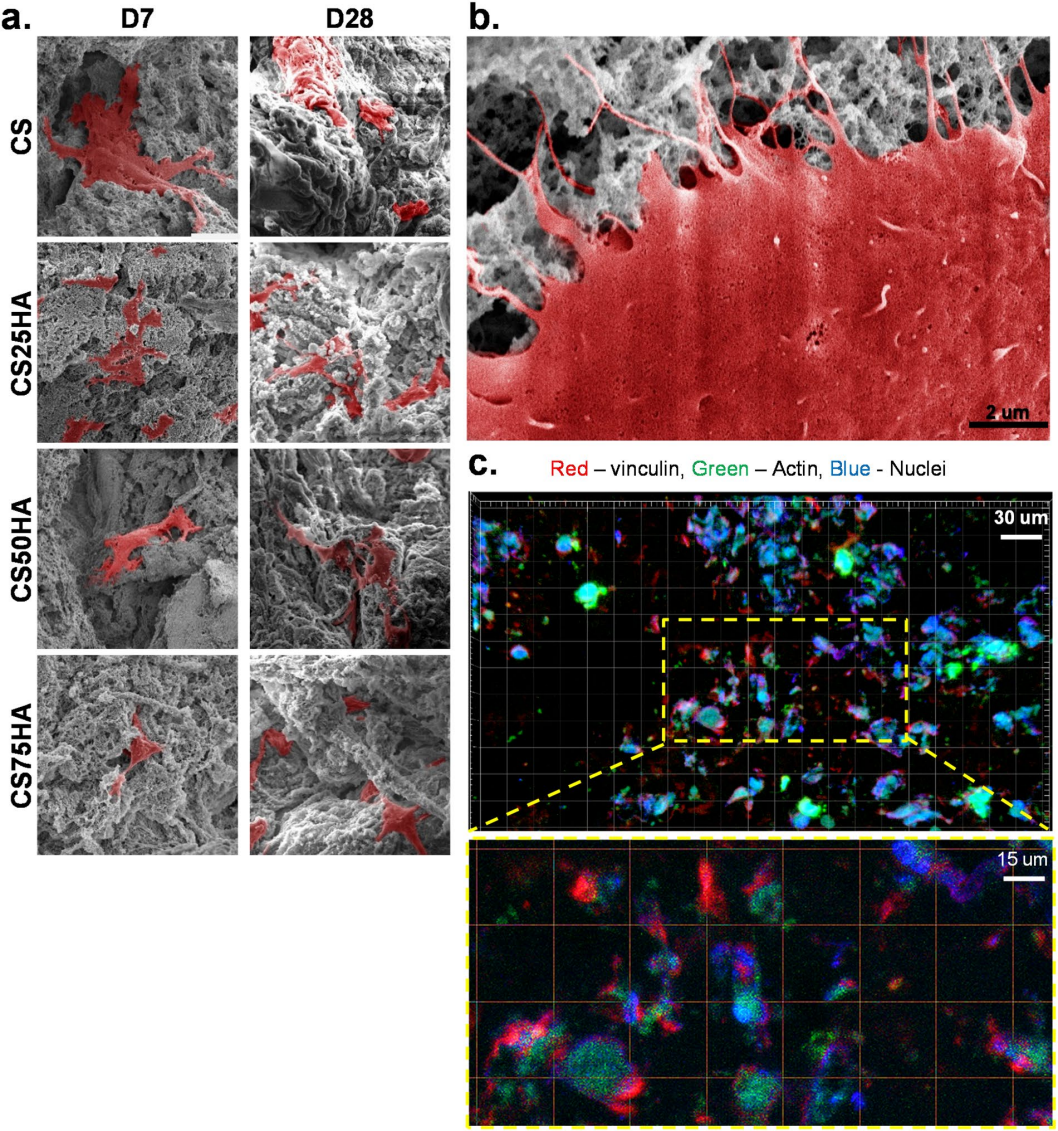
(**a**) SEM micrographs of MC3T3-E1 cells growing on purine-crosslinked chitosan scaffolds: CS, CS25HA, CS50HA, and CS75HA at days 7 and 28, cells are pseudo-coloured in red; Mag.: 5000X and (**b**) magnified micrograph representing the cellular attachment of MC3T3-E1 cells. The filopodia are shown infiltrating the scaffolds, Mag.: 20 000X, scale bar = 2 μm. (**c**) Immunofluorescence staining of vinculin (red), actin (green) and DAPI (blue) showing the attachment of the cells onto the scaffolds’ surface. Mag.: 40X, scale bar = 30 μm.

The morphology and attachment of the MC3T3-E1 cells grown in the maintenance medium over four weeks were assessed by SEM. Figure 2a shows the micrographs on day 7 and day 28. On both time points, cells in all composite scaffolds displayed a spread morphology and distinct, well organized F-actin fibres, characteristic of proper cell adhesion. The cell processes were found to infiltrate the pores of the scaffold (Fig. 2b). These SEM findings suggesting cell attachment to the scaffolds were further validated by fluorescently immunostained vinculin, a focal adhesion molecule associated with cell-to-cell and extracellular matrix (ECM) adhesion (Fig. 2c).

## Scaffolds with HA and PPase showed higher Osx expression

The presence of osterix (Osx/SP7), a key transcription factor required for osteoblast differentiation, in the nuclei of the encapsulated cells in chitosan alone, or with PPase or HA (two different concentrations: 3 mg/ml and 4.5 mg/ml) was tested (see Table 2).

**Table 2:** Concentrations for differentiation and mineralization study.

Group	Abbr	Chitosan (mg/ml)	GDP (mg/ml)	Hydroxyapatite (mg/ml)	Pyrophosphatase (units/mg chitosan)
Chitosan/PPase	CSP	6	100	0	0.67
Chitosan/50HA/PPase	CS50HAP	6	100	3.0	0.67
Chitosan/75HA/PPase	CS75HAP	6	100	4.5	0.67

**Figure 3:**
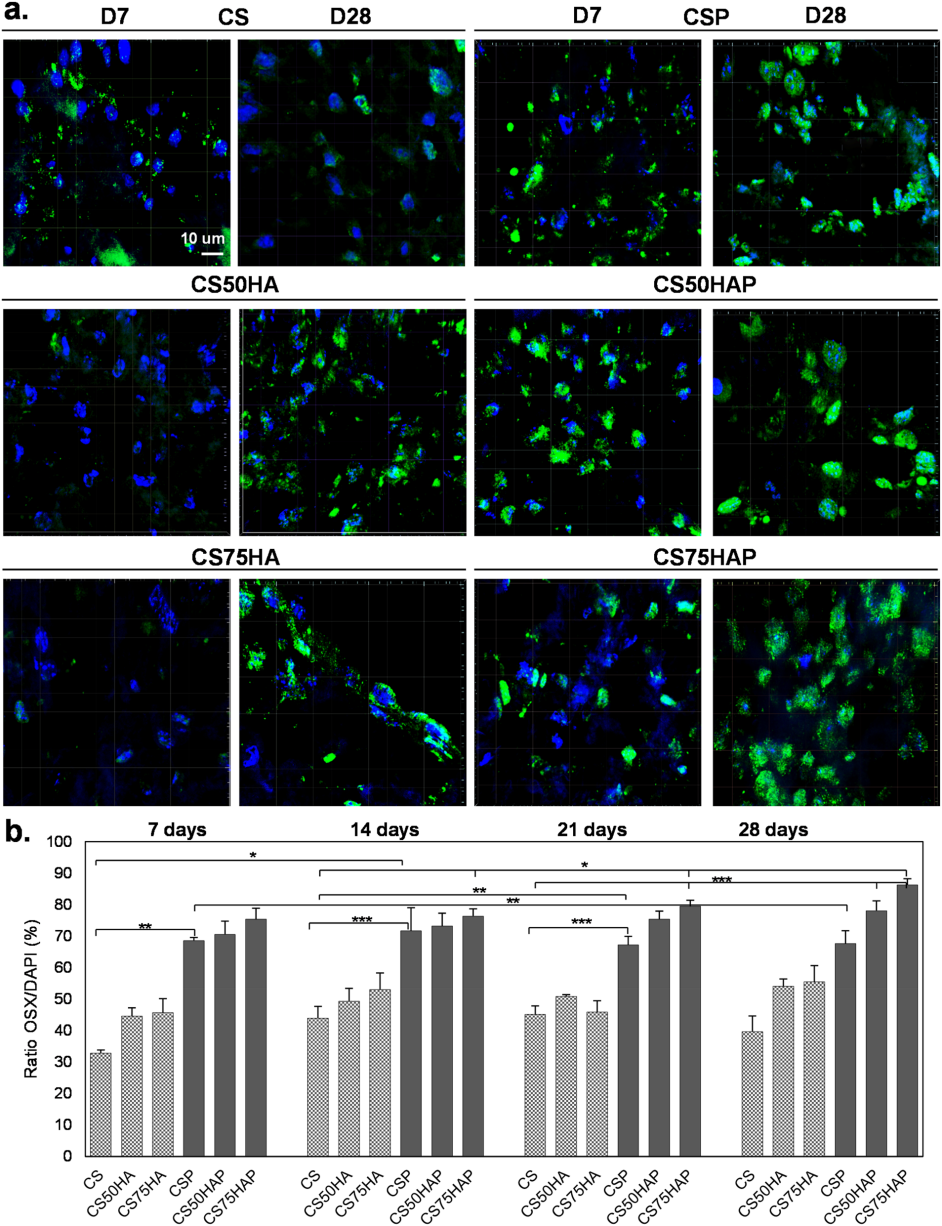
(**a**) Immunohistochemical analysis of frozen sections of scaffold-encapsulated MC3T3-E1 cells immunolabeled with Osterix/Sp7 (green) and counterstained with DAPI (blue), Mag.: 40X, scale bar = 10 μm. (**b**) The ratio of Osx/DAPI levels was quantified based on the colocalization of the two stains inside the nuclei versus nuclei with DAPI alone. *p* < 0.001(***), *p* < 0.01(**) and *p* < 0.01(*).

Cells grown for 7 or 28 days in the differentiation medium were sectioned using a cryostat and stained using an anti-Osx antibody. Fluorescence-confocal microscopy showed a noticeable increase in the nuclear accumulation of Osx in the groups containing PPase when compared to the control groups without PPase (Fig. 3a). Counting of total DAPI and Osx-stained nuclei showed that Osx was present in 30% of MC3T3-E1 cells in the control CS group compared to 70% in CSP and 80% in CS75HAP after seven days. The number of Osx-positive nuclei increased in the CS75HAP group throughout weeks 2, 3, and 4 (from 80–90%), when compared to that of CS after two and three weeks (Fig. 3b).

## Scaffolds containing PPase showed higher ALP activity

**Figure 4:**
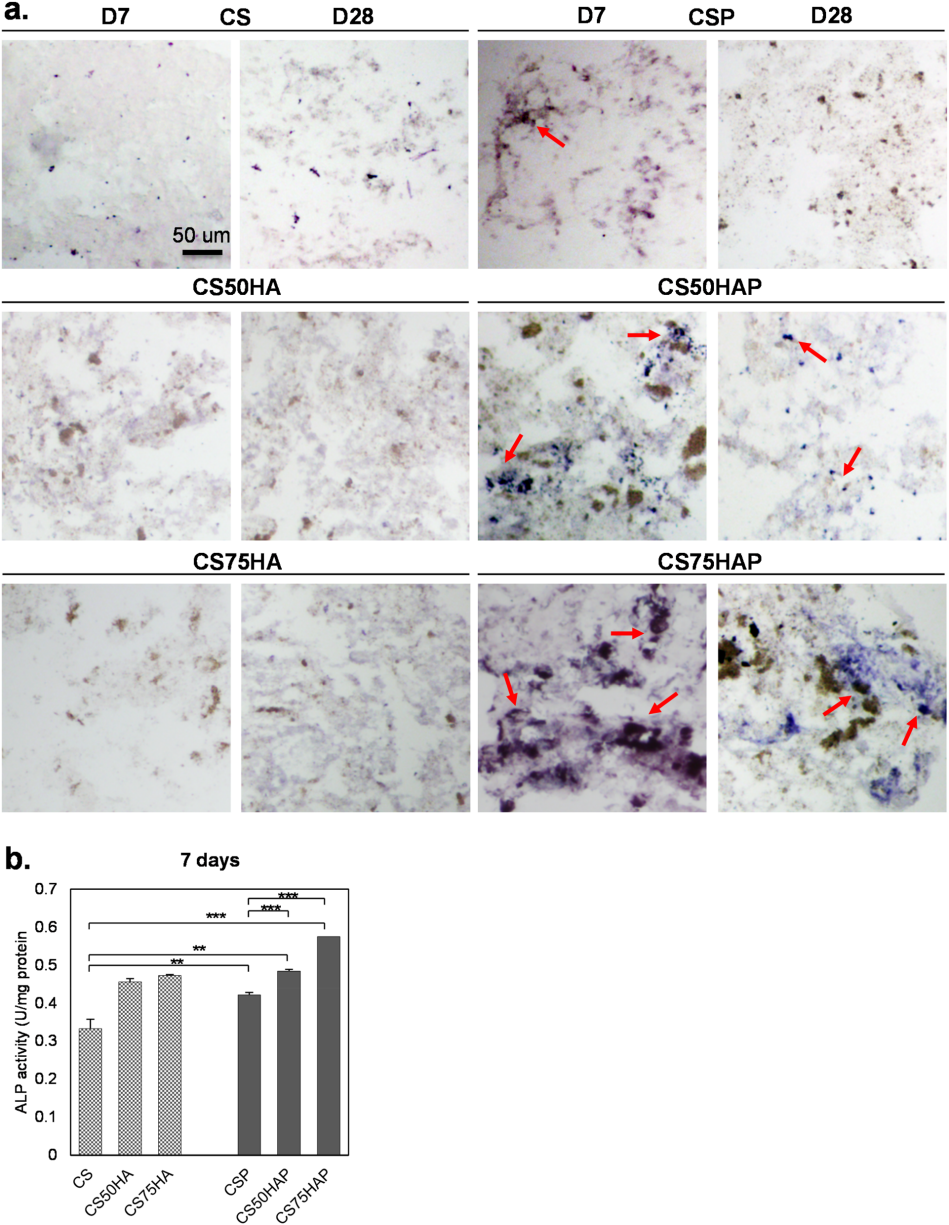
(**a**) Histochemical analysis of frozen sections of scaffold-encapsulated MC3T3-E1 cells (arrows) stained with BCIP/NBT for ALP activity (blue purple). Mag.: 20X, scale bar = 50 μm. (**b**) ALP activity was measured from the lysate of scaffold-encapsulated MC3T3-E1. The levels of ALP were normalized to total protein content. *p* < 0.001(***) and *p* < 0.01(**).

To further investigate the osteogenic differentiation in the encapsulated MC3T3-E1 cells, ALP activity was assessed by staining the cryosections of various groups of scaffolds (Fig. 4a). Seven days post-encapsulation, cells in the PPase containing scaffolds displayed more ALP staining than in the scaffolds without PPase. ALP activity was more pronounced in cells encapsulated in CSP, CS50HAP, and CS75HAP scaffolds than scaffolds without PPase; with CS75HAP showing the most intense staining for up to 2 weeks. On day 28, marked ALP activity was detected only in the CS75HAP group. These observations were further confirmed by spectrophotometric quantifications of ALP activity (Fig. 4b); there was a significant increase of ALP activities for the groups with both PPase (*p* < 0.01) and HA (*p* < 0.0001) and their combination (*p* < 0.0001).

## Scaffolds with HA and PPase showed higher deposition of minerals

**Figure 5:**
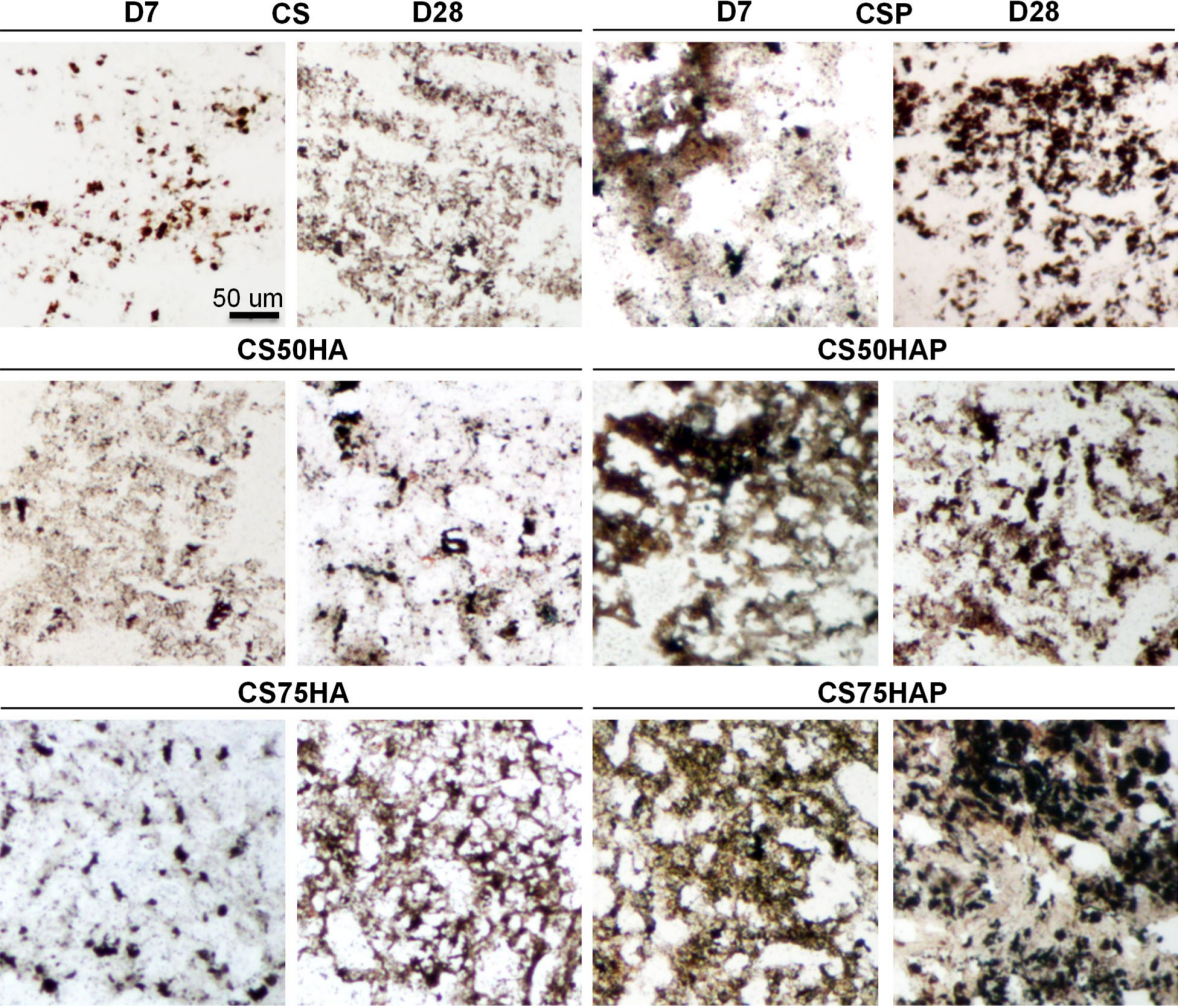
Detection of mineral deposition visualized by von Kossa staining (black) in frozen sections of MC3T3-E1-encapsulated scaffold at days 7 and 28 of differentiation. Mag.: 20X, scale bar = 50 μm.

The mineral deposits were investigated by staining the cryosections of cell-laden scaffolds following the method described by von Kossa. Black mineralized nodules were observed by light microscopy (Fig. 5). Once again, scaffolds with PPase showed a significantly increased deposition of minerals on day 28. Note that no visible difference was observed on day 7 in any of the groups without PPase.

## Implanted scaffold with HA and PPase resulted in higher bone and tissue volume

To test the in vivo osteogenic properties of the scaffold, the cell-free CS75HAP scaffold was used in a rod-fixated tibia fracture surgery on 4-month old mice. The choice of this scaffold was based on the fact that it resulted in the highest osteogenic effects (highest osteoblast number, Osx expression, ALP activity, and mineralization) in vitro. Both cell-free CS and CS75HA scaffolds were used as controls in separate groups of mice undergoing fracture surgery. The SHAM group included the mice with a tibial fracture without scaffold insertion. Fracture healing in all these groups was examined by micro-CT and histological analyses at 17 days post-surgery.

**Figure 6:**
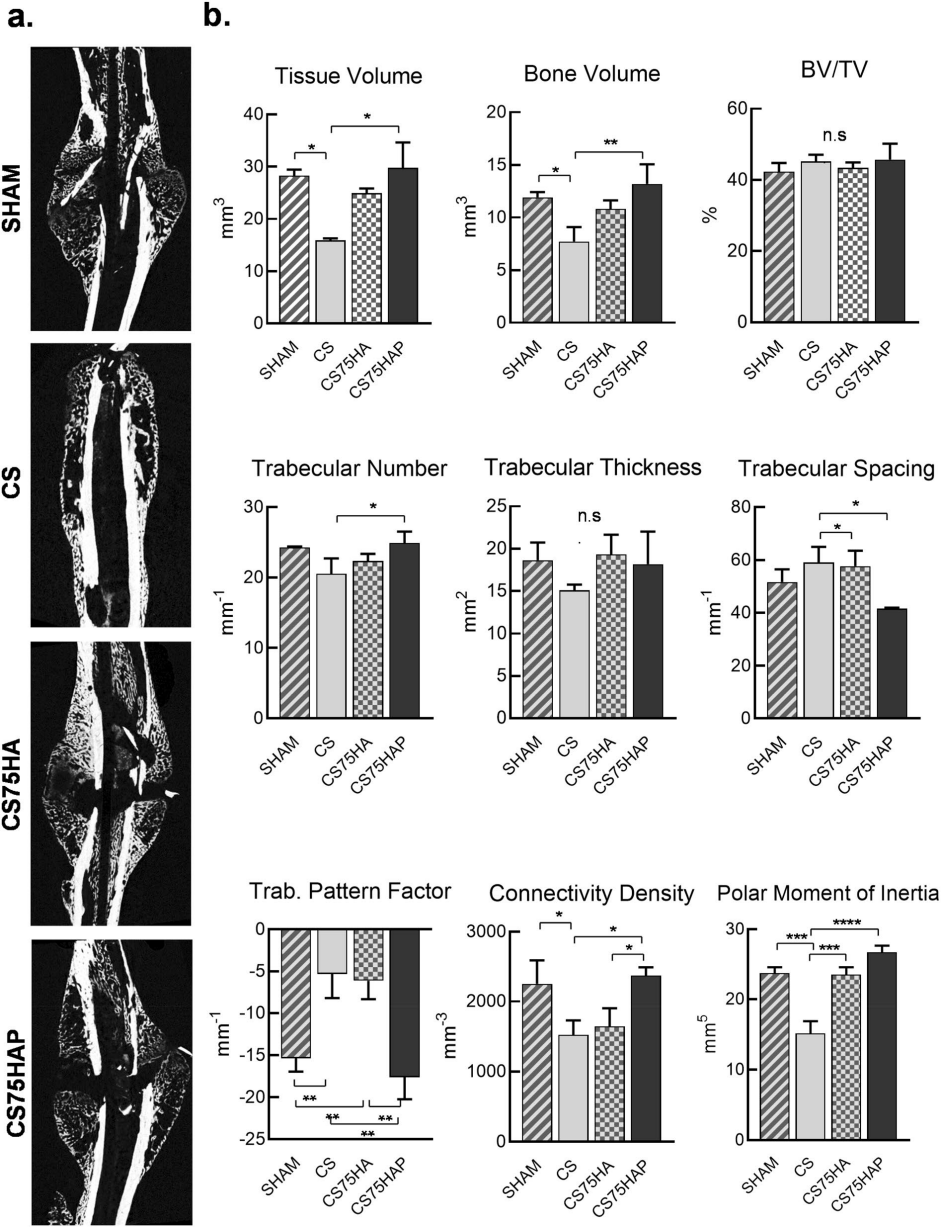
(**a**) Micro-CT images showing the morphology of the callus at day 17 post-surgery for each group: SHAM, CS, CS75HA, and CS75HAP. (**b**) Tissue volume, bone volume, trabecular number, trabecular spacing, trabecular pattern factor, connectivity density, and polar moment of inertia all displayed some significant differences among the groups. Bone volume density (BV/TV) and trabecular thickness were not significantly different between the groups. *p* < 0.0001(****), *p* < 0.001(***), *p* < 0.01(**) and *p* < 0.01(*).

Micro-CT scans revealed a significant increase of total bone volume at the fracture sites in the CS75HAP group in comparison to the control CS group (Fig. 6a). This increase of total bone volume was accompanied by a significant increase of total tissue volume in the callus of the CS75HAP as well. Although not statistically significant, total bone and tissue volumes in the CS75HA group also showed a tendency to be higher than the CS group.

Interestingly, SHAM and CS75HAP groups showed comparable total bone and tissue volumes at the callus, while in both cases, these values were significantly higher than the CS group. When bone volumes were normalized by tissue volumes, no significant difference in BV/TV was observed among all different groups compared. When the trabecular measurements were compared, CS75HAP had a significantly higher trabecular number than the CS group. SHAM and CS75HAP were both significantly higher than CS in terms of trabecular spacing. No significant differences were reported for the trabecular thickness among the four groups. The trabecular bone pattern factor shows higher numerical values for SHAM and CS75HAP when compared to CS and CS75HA. A higher connectivity density was also observed in SHAM and CS75HAP when compared to CS and CS75HA. Lastly, CS had a lower polar moment of inertia than all other groups (Fig. 6b).

**Figure 7:**
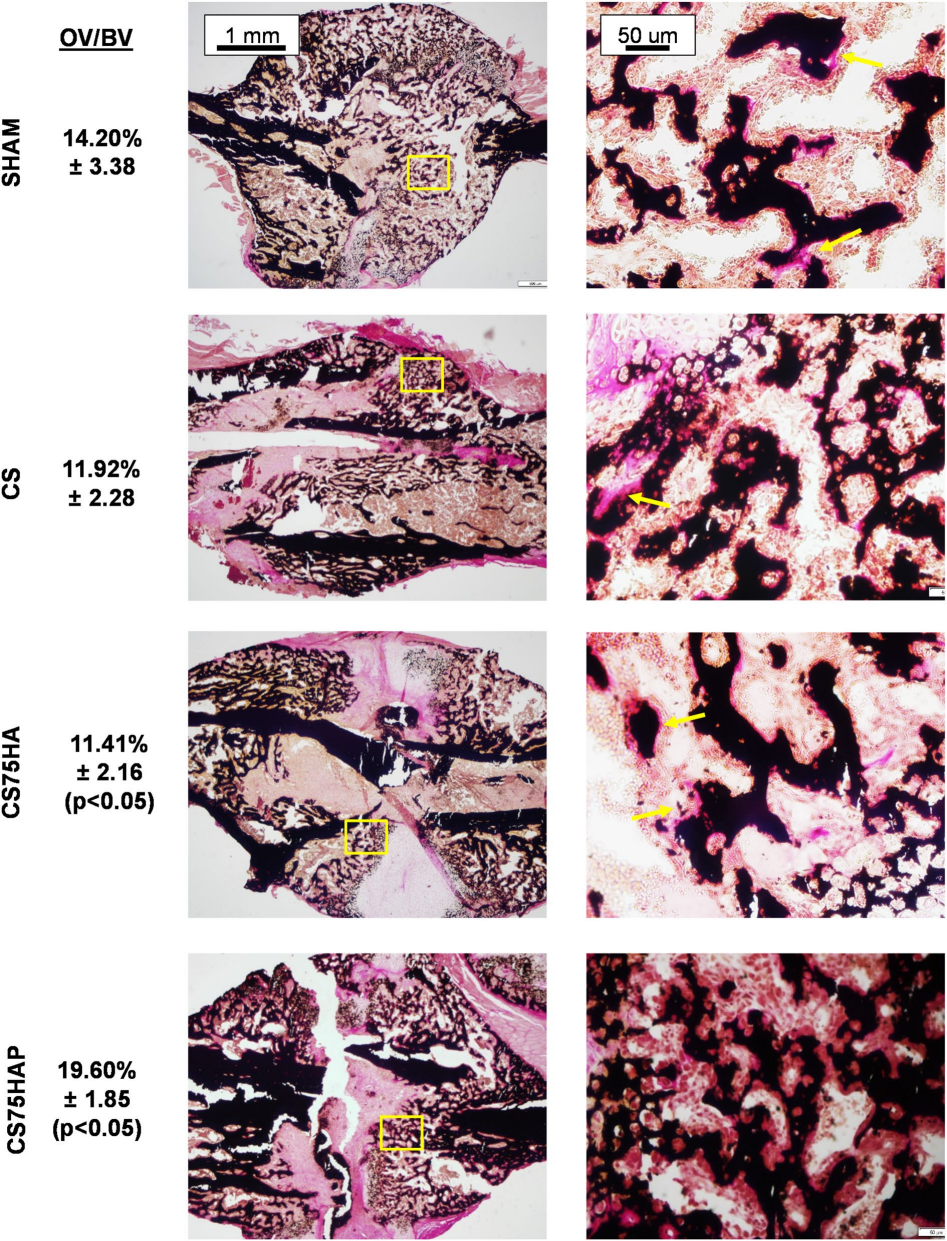
Histological sections of callused tibiae from 4-month old WT mice 17-days post-surgery. The samples were stained with VK/VG at low and high magnification showing osteoid formation (arrows). Quantification of osteoid volume/bone volume (OV/BV) among the four groups is indicated. Lower Mag.: 5X, scale bar = 1 mm; Higher Mag.: 20X, scale bar = 50 μm.

**Figure 8:**
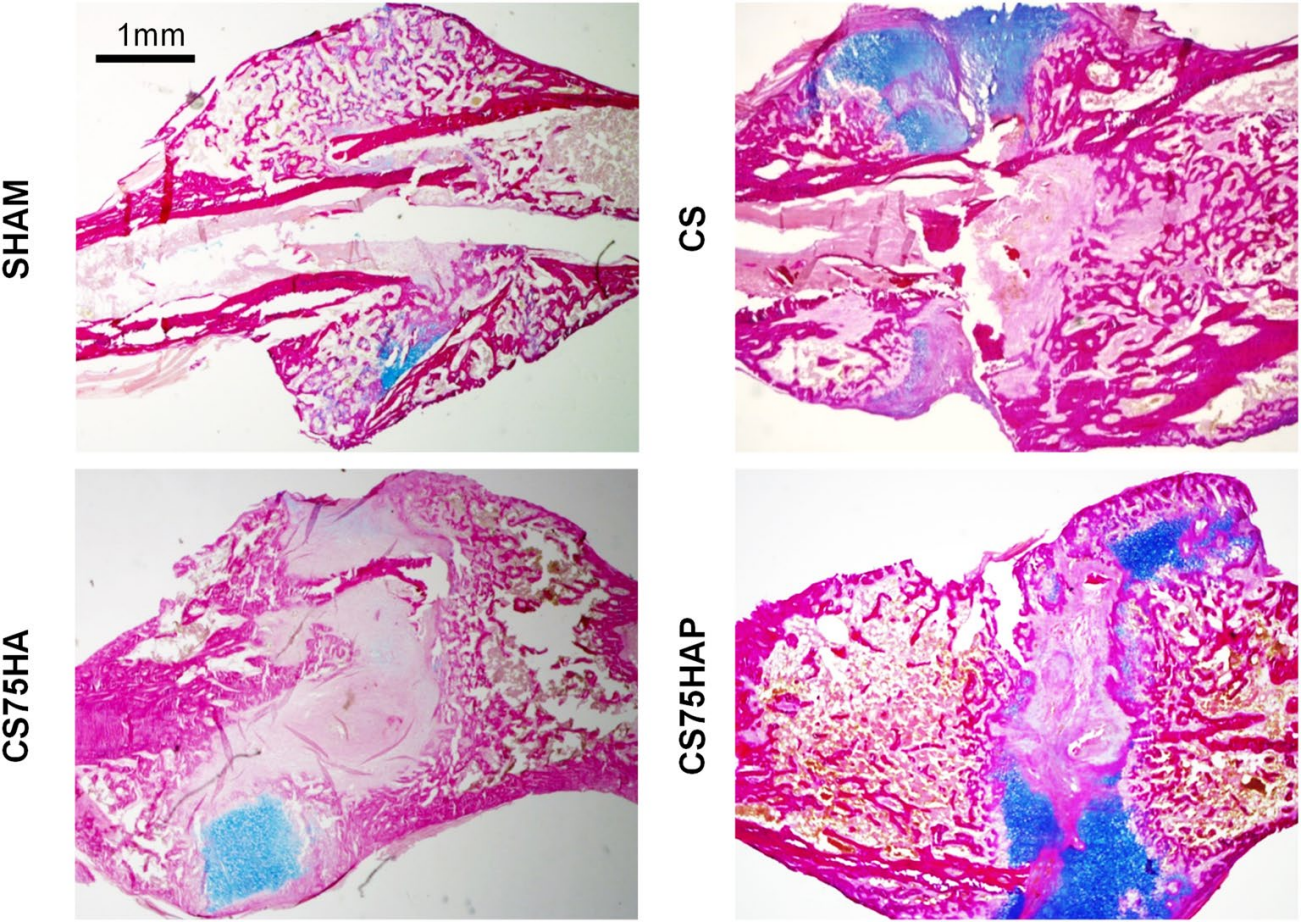
Histological sections of callused tibiae from 4-month old WT mice 17-days post-surgery. The samples were stained with AB/VG at low and high magnification showing cartilage matrix (blue). Mag.: 5X, scale bar = 1 mm.

Next, to assess the effect of the scaffolds on the mineralization of the newly synthesized ECM (osteoid) in the callus, the sections of the plastic embedded fractured bones were stained by von Kossa and van Gieson stains and examined by light microscopy. Significantly higher amount of osteoid was observed in CS75HAP group among all three groups with implanted scaffolds. However, no significant difference was observed between CS75HAP group and the SHAM group (Fig. 7). To investigate the effects of the scaffolds on the cartilage-to-bone transition at the fracture sites, decalcified bone sections were stained by Alcian Blue and van Gieson. Histological analyses indicated the presence of cartilage matrix in all groups in a comparable manner (Fig. 8).

## Discussion

Bone tissue engineering commonly involves the use of a scaffold that will act as a carrier of bioactive agents to promote bone cell functions and induce new bone formation. Several strategies have been proposed to improve the efficacy of bone regeneration by making it faster with coordinated regulation of the sequential anabolic steps involved in the process. It is a routine practice to use a combination of biomaterials and pro-osteogenic molecules that can stimulate osteoblast differentiation, proliferation, and function. In this study, we carried out a thorough in vitro and in vivo investigation of a new formulation of purine nucleotide (GDP)-crosslinked chitosan-based composite scaffold to test the effects of its two components, i.e. HA bioceramics and PPase, on cell behaviour and biomineralization.

Chitosan solutions with HA and PPase were ionically crosslinked with a GDP solution containing MC3T3-E1 pre-osteoblasts to form a cell-laden 3D scaffold (CS75HAP). This composite biomaterial has been shown to be biocompatible and bioactive when used for bone regeneration^24,38^. The purpose of incorporating these components in the GDP-crosslinked chitosan scaffold was to obtain a rapidly gelling scaffold with increased osteogenic capability. The proof of concept for a 3D bone scaffold was attained through pre-osteoblast cell encapsulation studies, which also led to identifying the CS75HAP as the most pro-osteogenic scaffold. By adapting a fracture healing model to mice^39^, the effects of CS75HAP on the cell, and tissue properties in the fracture callus at the mid-point of the healing process were found to be superior as compared to CS and CS75HA. This superiority was associated with the presence of the PPase in the formulation of the scaffold.

It has been shown that GDP in the chitosan scaffold can be hydrolysed into inorganic phosphate (P_i_) and guanosine monophosphate (GMP) overtime by cellular GTPase activity^40^. Alternatively, GDP can be enzymatically cleaved into PP_i_ and guanosine^41^. P_i_ and PP_i_ generated by the scaffold may differentially affect the process of mineralization of the newly synthesized collagen ECM. While higher P_i_ levels in the extracellular fluid promotes the precipitation of calcium phosphate minerals both within and outside the collagen fibres, incorporation of PP_i,_ into the nascent calcium phosphate crystals prevents crystal growth^33^. PP_i_ has been known as an inhibitor of ECM mineralization for a long time^42^. Many metabolic reactions generate PP_i_ intracellularly, which is pumped out into the extracellular space via a transmembrane protein ANK^43^. Also, ectoenzymes present outside the cells produce PP_i_ from different nucleotides^44^. The role of PP_i_ as a potent inhibitor of ECM mineralization has been confirmed by genetic mutations in humans and animal models with reduced extracellular PP_i_ levels leading to severe soft tissue calcification^45–47^. Interestingly, although PP_i_ is produced in bone, it is cleaved to P_i_ by ALP produced by osteoblasts^48^. While designing our scaffold, this aspect of osteoblast biology was mimicked through the incorporation of PPase in the scaffold formulation. PPase breaks down mineralization inhibitor PP_i_ generated as a breakdown product of the chitosan scaffold into two P_i_ ions, thereby converting the mineralization inhibitor to mineralization promoting ions.

In addition to analysing cell viability/proliferation and attachment inside the scaffolds, the differentiation ability and functional properties of the encapsulated cells were assessed by the number of Osx-positive cells in various scaffolds. While both Osx-positive and Osx-negative cells were observed in the scaffolds incubated in the differentiation medium, the total cell number, as well as the relative percentage of Osx-positive cells, were higher in the CS75HAP scaffold; this suggests that the novel biomaterial has promoted both cell viability/proliferation and differentiation. Accordingly, the highest total ALP activity in the cells in the CS75HAP scaffold was obtained. Furthermore, more intense ALP staining was observed on the frozen sections of the CS75HAP scaffold in comparison to that of the control scaffold, indicating that more cells differentiated to functional osteoblasts in the former. This inference was further confirmed by the highest amount of calcium phosphate minerals deposited within the CS75HAP scaffold. This differed from some composite chitosan scaffolds that did not show any difference in ALP activity between the calcium phosphate composite and the chitosan scaffold alone^49^.

We used a rodent fracture healing model to examine the effects of CS75HAP during the early stages of the healing process. Fracture healing is a complex process that takes place in three distinct stages: the initial inflammatory stage, the reparative stage, and the remodelling stage. The reparative stage can occur via two mechanisms: direct healing, which occurs without a cartilaginous intermediate and indirect bone healing where there is formation of a cartilaginous callus^50^. Indirect fracture healing is the most common form of repair and consists of both endochondral and intramembranous ossifications^51,52^. Therefore, in the current study, we used an intramedullary nailing procedure to introduce tibial fracture as this procedure results in indirect fracture healing involving endochondral ossification as the predominant process for bone regeneration. The fracture site in this model first forms a soft callus and subsequently a hard callus. This well-established protocol of orthopaedic surgery has formerly been used successfully in mice to study osteogenesis during fracture healing^39,53^.

Our in vivo studies confirmed that the CS75HAP scaffolds induce enhancement in bone formation, detected both by μCT and histological analysis of the callus area 17 days post-implantation, when compared with animals implanted with CS or CS75HA scaffolds alone. Both the quantitative parameters (total tissue and bone volume) and the architectural parameters showing porosity and complexity provided an accurate indication of the structural changes of the callus associated with the healing progression^54^. Notably, a higher trabecular bone pattern factor in the CS75HAP group indicates a highly porous structure, e.g. a higher amount of newly mineralized callus ECM. Given the age of the mice (4 months) and the stage at which the callused tibia was examined (17 days), the findings from the SHAM and the CS75HAP group seem consistent with published results of fracture healing in rodent models^55,56^. They both showed a highly porous callus with a higher volume, larger trabeculae, and a bigger polar moment, which translates to mechanical superiority. Comparatively, these showed better results than a chitosan/HA porous scaffold that was found to be still present inside a tibial rat fracture after 15 days of implantation^57^.

In general, an increase of bone anabolic activities at the fracture site results in the deposition of a higher amount of unmineralized ECM (osteoid)^53^. Although this is true for both SHAM and CS75HAP groups, CS and CS75HA groups did not show an increased deposition of osteoid. Together with the observation that total bone volume is lower in these two latter groups, this may suggest a slower rate of bone formation at the fracture site of these two groups. It is interesting to note that although CS and CS75HA scaffolds did not carry PPase, there was no visible presence of unmineralized osteoid at the fractures with these composites. An increase of osteoid volume was expected due to the anticipated local accumulation of PP_i_ released from the scaffolds without the PPase activity. However, it is possible that high expression of endogenous ALP by osteoblasts, which non-specifically cleaves PP_i_, might have reduced the local PP_i_ levels to facilitate the mineralization of the unmineralized osteoid^48^. On the other hand, despite the presence of PPase in CS75HAP, there was a higher amount of osteoid at the fracture with this composite in comparison to CS and CS75HA. This increase of osteoid amount can be explained by an increase of new ECM synthesis, which accumulates before it is fully mineralized. If this is indeed the case, it will be interesting to investigate in the future how the inclusion of PPase to CS75HAP leads to a higher bone synthesis in comparison to the CS75HA group.

The key focus of this study was to investigate the biocompatibility and osteogenic potential of our newly formulated injectable crosslinked chitosan scaffolds. The osteogenic effects of chitosan (with or without HA) in vitro and in vivo have been described in the literature^58–61^. Our findings agree with these studies and proceed to show enhanced osteogenic effects of CS75HAP in vitro. We performed cell culture studies to establish the biocompatibility of this biomaterial as well as a positive effect on the osteogenic differentiation of MC3T3-E1 cells in the presence of HA and PPase molecules within the scaffold. Among the tested scaffolds, CS75HAP showed the best biocompatibility and the normal initiation of tibial fracture healing 17 days post-implantation in vivo. The presence of new bone in the callus of the tibia suggests a better osteogenic potential of this scaffold. Considering that fracture healing with CS75HAP is comparable to that of SHAM operated group, further modifications such as encapsulation of MSCs in combination with pro-osteogenic molecules can be made to achieve improved experimental outcomes.

Some limitations of this study are small sample size, analysis at a single time point after the fracture, and lack of mechanical testing, which need to be addressed in future studies. The material can also be tested using different animal models, e.g., model with a critical-sized bone defect, which is another suitable model to study bone repair^22,62^. Also, the size of the implanted scaffold might have to be revised in the future. Finding an optimal size of scaffold is essential to prevent it from acting as a barrier for cell–cell interactions, which may hinder the healing process. Also, longer time points are required to study later stages of fracture healing. Further studies will be needed to address these questions.

## Conclusion

Among several chitosan-based injectable composite scaffolds, our study identifies a novel scaffold that appears to be superior both in vitro and in vivo. This novel biomaterial can be used as a base material for further improvisation, which can be tested in bone repair applications in the future. In the long run, our work may pave the way for the development of minimally invasive procedures using injectable osteogenic biomaterials for faster repair of injury-induced bone defects.

## Methods

### Materials

Chitosan was purchased from MP Biomedicals (USA), 100–300 kDa, 93.37% deacetylated. GDP (A2754), nanopowders of HA (< 200 nm, XRD assay conforms to structure, purity: 97%) and β-TCP (< 200 nm) were purchased from Sigma-Aldrich (USA). Inorganic yeast pyrophosphatase was purchased from New England Biolabs (USA). All items were used as received without any modification. All cell culture reagents (α-MEM, foetal bovine serum, and penicillin–streptomycin) were purchased from GIBCO, (USA). Alamar Blue solution, BCIP(R)/NBT liquid substrate system, and reagents for alkaline phosphatase (ALP) Assay were obtained from Invitrogen (USA), Sigma-Aldrich (USA) and Abcam (UK), respectively. The ALP levels were standardized to the protein content measured by Pierce™ BCA Protein Assay Kit (Thermo Fisher, USA). The focal adhesion staining kit was obtained by Millipore-Sigma (USA). Anti-Sp7/Osterix antibody and Goat anti-rabbit IgG H&L (Alexa Fluor® 488) were purchased from Abcam (USA). The Quincke BD 25-gauge 3″ spinal needle (450,170) was purchased from Becton Dickinson (USA).

### Mice

Sixteen 2-month-old, male wild type C57BL/6 J mice were purchased from the Jackson Laboratory (stock number 000664). . Mice were housed in standard cages under a 12-h light/dark cycle. All animal procedures were reviewed and approved by the McGill Institutional Animal Care and Use Committee following the guidelines of the Canadian Council on Animal Care.

### Preparation of apatite-chitosan soft scaffold

Scaffolds were made following a protocol developed for the preparation of the chitosan scaffold using GDP^63^ and apatites^64^. The chitosan/HA solutions were made at pH 5 and left under stirring for 4 h at room temperature. Freshly prepared GDP solution was added to the chitosan/HA suspension. The protocol was modified by the addition of MC3T3-E1 cells to the GDP solution and 1 unit of pyrophosphatase (10 uL) in the chitosan/HA solution right before mixing the solutions (Supplementary Figure S1). The resulting scaffolds were spherical in shape, with a diameter of 5 mm, and volume of approximately 0.065 mm^3^. To ensure the sterility of the scaffolds, the HA powder was autoclaved, and both the chitosan and GDP solutions were filtered using 0.22 μm syringe filters.

### Cell culture

The cell culture methodology was the same as previously described^64^. Briefly, MC3T3-E1 pre-osteoblasts were grown in proliferation medium (α-MEM + 10% foetal bovine serum + 100 units/ml of penicillin and 100 µg/ml of streptomycin. At 75% confluency, cells were trypsinized and encapsulated in the scaffolds at a density of 10^7^ cells/scaffold. The differentiation medium (α-MEM medium supplemented with 2.16 mg/ml β-glycerophosphate and 50 μg/ml ascorbic acid) was prepared as described by us previously^32^. The cell-laden scaffolds were rinsed twice in fresh culture medium, and cultured in the same medium at 37 °C.

### Cell proliferation and attachment assessment

Cells were encapsulated and maintained in proliferation medium for 7, 14, 21, and 28 days, after which the scaffolds were rinsed three times in Dulbecco's phosphate-buffered saline without calcium or magnesium (DPBS^-^) (Gibco, 14,190–144). Then the samples were fixed in 4% paraformaldehyde in DBPS^-^ for 20 min at room temperature (RT) and permeabilized in a 0.2% Triton-X-100 detergent for 3 min. 5% bovine serum albumin (BSA) was used to block against non-specific antigen-binding before incubating the samples in Alexa Fluor® 647 Phalloidin for the cytoskeleton (Molecular Probes, A22287, 1/1,000) for 1 hr at RT and DAPI for the nucleus (Molecular Probes, D1306, 1/5,000) for 20 min^64^. The same was done with Vinculin monoclonal antibody, purified clone 7F9 (1/2000), followed by Goat Anti-Rabbit IgG H&L (Alexa Fluor® 488) to assess cell attachment. Images were collected (LSM710 Confocal Microscope, Zeiss, Germany) and image processing and analysis were completed (Imaris Software) in the McGill University Life Sciences Complex Advanced BioImaging Facility (ABIF). At least three independent experiments were performed in this study, with an n = 3 for each sample.

### Cell viability assessment

Cells were encapsulated in the scaffolds as described above. For negative control, an empty scaffold was used. The cells were grown in proliferation medium for 28 days and were assessed for viability every 3 days, starting from day 1. At each time point, the cell-laden scaffolds were incubated with alamarBlue for 4 h at 37 °C, after which the supernatant’s change in colour was detected by absorbance at 570nm^64^. This assay was done repeatedly on the same cell-laden scaffolds over the four weeks. At least three independent experiments were performed in this study, with an n = 3 for each sample.

### Cell morphology assessment by scanning electron microscopy

The cell-laden scaffolds were maintained in proliferation media for 7 and 28 days. The samples were first fixed in 4% paraformaldehyde and then dehydrated in serially increasing concentrations of ethanol, followed by increasing concentrations of amyl acetate, and then dried in a critical point dryer (CPD) (Leica CPD 030, Germany)^64^. Then, the scaffolds were coated with 5 nm platinum and examined by field emission scanning electron microscopy (High voltage: 5 keV, Spot: 2.0; FE-SEM, Inspect F50, FEI, USA)^64^. The pseudo-coloring of the images was done using Lightroom (Photoshop).

### Cryosections

The cell-laden samples were maintained in differentiation medium for 7, 14, 21, and 28 days and embedded in TissueTek freezing medium for cryosectioning (the procedure is showed in Supplementary Table S1). Frozen blocks were sectioned using a cryostat (CryoStar™ NX70 Cryostat) at − 25 °C. For each block, 7-μm cross-sections of the samples were collected on SuperFrost® Plus Blue slides (Fisher Scientific). The slides were air-dried for several hours before Von Kossa, ALP, and Osterix/DAPI staining.

### Encapsulated cell staining and immunohistochemistry

Von Kossa staining for mineral deposition was performed on the cryosections as described^65^. ALP staining of the cryosections was achieved by adding 50 μL of BCIP(R)/NBT solution on each sample for 30 min. The slides were then washed in deionized water and mounted with Aqueous Mounting Medium (Bio-Rad, USA). For both von Kossa and immunohistochemistry, the cryosections were boiled in antigen retrieval buffer for 10 min, then blocked with 5% bovine serum albumin (BSA; Fisher, Pittsburgh, PA) in Tris-buffered saline (TBS)–Triton X-100, followed by incubation with a rabbit anti-osterix antibody. Localization of the primary antibody was detected with a fluorescent-labelled goat anti-rabbit IgG. After three washes with PBS, the nuclei were visualized with DAPI. Images were captured by the LSM710 confocal microscope.

### ALP assay

Cell extracts were prepared with 1X Passive Lysis Buffer (Promega, Madison,WI) and total proteins were measured by the MicroBCA protein assay. ALP activity was measured using ρ-nitrophenyl phosphate substrate (Sigma-Aldrich, Saint Louis, MO) and then normalized by the respective protein concentration in the extracts.

### In vivo testing of the scaffold in a rod-fixated tibia fracture

**Figure 9:**
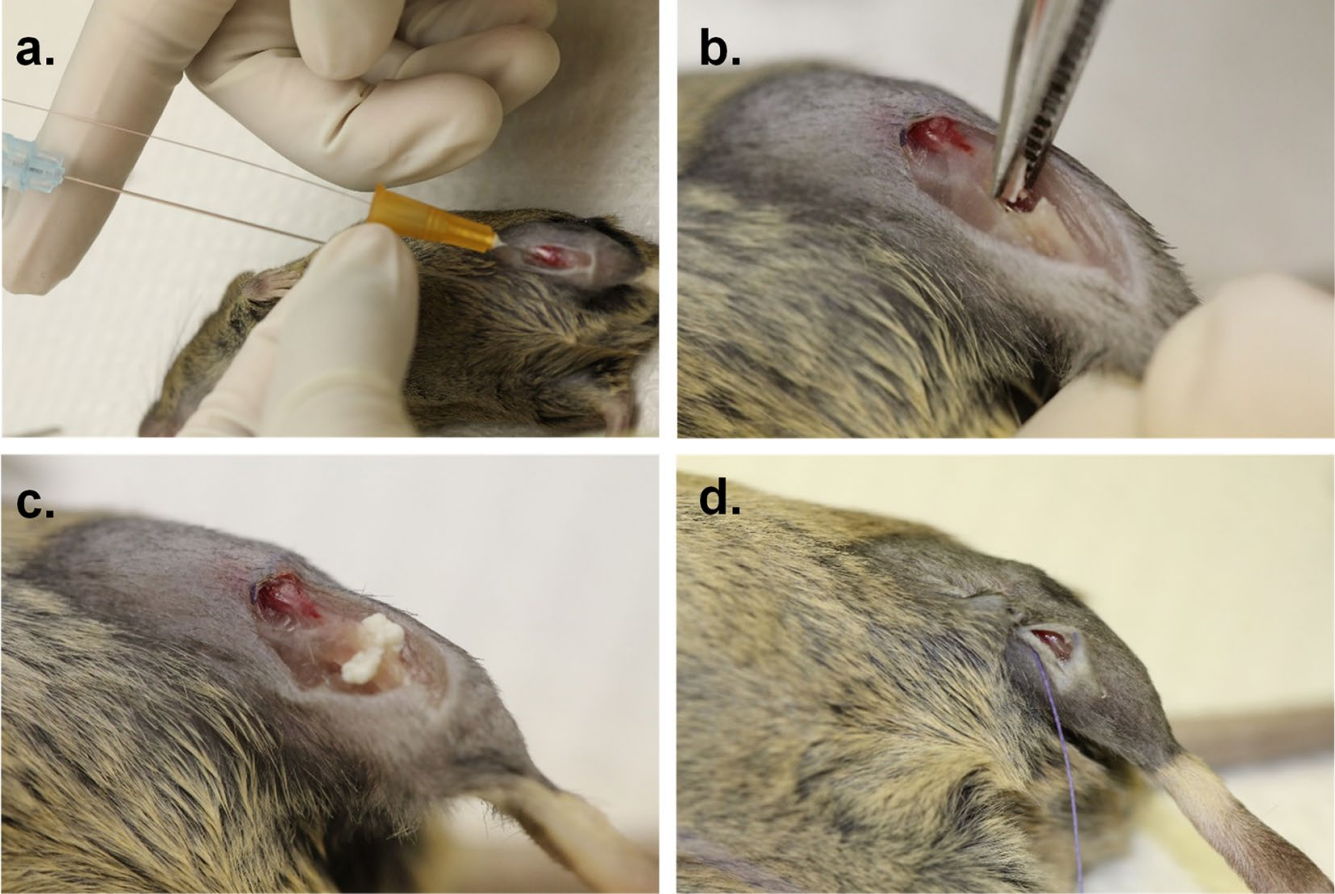
Rod-fixed tibial fracture surgical procedure in mice. (**a**) a rod is inserted in the intramedullary canal of the tibia through a parapatellar incision (**b**) a mid-tibial shaft osteotomy is made using a pair of extra fine Bonn scissors. (**c**) The scaffold is injected at the fracture site. (**d**) The incision was sutured with a single 6–0 vicryl suture.

The intramedullary nailing surgery was completed on the right tibia at four months of age. The analyses of the fractured bones were performed 17 days post-surgery. The surgical procedures were done as previously described^66,67^. The mice were first injected subcutaneously with 0.04 ml of buprenorphine (0.3 mg diluted in 12 ml of normal saline) before the surgery. Then, the mice were anesthetized using isoflurane gas and oxygen and kept under anaesthesia through the full length of the surgery. Once mice were anesthetized, they were injected with carprofen (5 μl/g) and normal saline (20 μl/g). The right leg was then shaved to remove all fur surrounding the tibia. The surgical site was prepared with 2% chlorhexidine and 70% ethanol. A 3-mm vertical skin incision was made above the knee, and the patellar ligament was exposed where a vertical medial parapatellar incision (about 1–1.5 mm in length) was made on the tibial plateau. A 25-gauge needle was inserted through the incision into the tibial canal and through the needle, the internal wire core guide was inserted. The 25-gauge needle was then removed whilst keeping the internal wire core guide in the tibial canal. The protruding segment of the wire core guide was cut and bent at 90° at the proximal end and tucked in under the patellar ligament to avoid any puncture. Using the same skin incision, a mid-tibial shaft osteotomy was made using an extra fine Bonn scissors (14,084–08; Fine Science Tools, Vancouver, Canada). The procedure was then modified for the insertion of the scaffolds (Table 3) which were placed inside the fracture before the incision was sutured with a single 6–0 vicryl suture (Johnson & Johnson, Skillman, NJ) (Fig. 9). 1 to 2 drops of analgesia, lidocaine/bupivacaine, were added to the fracture site. The skin was sutured using three to four horizontal mattress sutures. After the surgery, the mice were placed in their cages on a heated pad and allowed to move around freely. The mice were then given subcutaneous injections of 0.05 ml of buprenorphine and carprofen (5 μl/g) at 24 and 48 h post-surgery. The animals were closely monitored, and any mice showing signs of severe pain, bleeding, or swelling were euthanized. All procedures were performed under aseptic conditions in the procedure room of the animal facility at the Shriners Hospital for Children, Montreal, Quebec, Canada.

**Table 3:** Concentrations for in vivo study.

	Abbr	Chitosan (mg/ml)	GDP (mg/ml)	Hydroxyapatite (mg/ml)	Pyrophosphatase (units/mg of chitosan)	# Mice
SHAM	SHAM	0	0	0	0	3
Chitosan	CS	6	100	0	0	3
Chitosan/HA	CSH	6	100	4.5	0	4
Chitosan/HA/PPA	CSHP	6	100	4.5	1.67	5

### Sample collection

The mice were sacrificed using isoflurane and CO_2_ at 17 days post-surgery. The tibiae were dissected at the level of the knee and ankle and prepared for micro-CT and histological analyses, as described previously^66^.

### Micro-CT

The SkyScan 1,272 micro-CT machine (SkyScan, Bruker, Belgium) was used to image samples. Tibiae were harvested and imaged in plastic tubes containing 70% ethanol. The scans were performed using the SkyScan 1,272 software as described previously^66^. The source voltage used was of 60 kV, with a source current of 166 A. The acquisition parameters were as follows: pixel size, 5.00 m; rotation step, 0.4; aluminium, 0.5-mm filter; and image size of 2,452 by 1,640 pixels. Then, NRecon Reconstruction Software was used with a beam-hardening correction of 15%, a ring artefact correction of 6, smoothing of 2, and a dynamic range of 0.00 to 0.12. The reconstructed images were then rotated using DataViewer and analysed using CTAn (both from SkyScan).

### Histology and histomorphometry

Tibias from all four groups were fixed overnight in 4% Paraformaldehyde, embedded in methyl methacrylate, sectioned (7-μm thickness), and processed for von Kossa and van Gieson (VKVG) staining. Stained bone sections were analysed for osteoid volume/bone volume (OV/BV) using the Osteomeasure software (Osteometrics, Inc.) at objective 40. The region of interest (ROI) was selected within the fracture healing site (5 random locations for each sample). The sections were also decalcified in a Cal-Ex decalcifier solution (Fisher, Pittsburgh, PA) overnight and processed for Alcian Blue and van Gieson (ABVG) staining. Images were taken at room temperature using a light microscope (DM200; Leica) with a 5, 20 or 40-objective. All histological images were captured using a camera (DP72; Olympus), acquired with DP2-BSW software (XV3.0; Olympus), and processed using Photoshop (Adobe).

### Statistical methods

GraphPad-Prism8 software was used to perform statistical analysis. One-way unpaired and two-way ANOVAs were used to analyse statistical differences between samples, and Tukey’s multiple comparison test was done to find which means were different from each other, at a confidence coefficient of 0.95. The Shapiro–Wilk test was performed to test normality. All error bars represent the standard deviation. The power for comparing significantly different means was calculated for each sample group and were all over 80% by the normal approximation method (OpenEpi website). Statistical differences were considered significant when *p* < 0.05. Asterisks represented the following *p* values: *p* < 0.0001(****), *p* < 0.001(***), *p* < 0.01(**) and *p* < 0.01(*).

## Supplementary information

Supplementary file1 (DOCX 180 kb)

## Data Availability

The data that support the findings of this study are available from Dr. Monzur Murshed, but restrictions apply to the availability of these data, which were used under license for the current study, and so are not publicly available. Data are however available from the authors upon reasonable request and with permission of Dr. Monzur Murshed.
