# Supplementary material for: In vitro and in vivo investigation of osteogenic properties of self-contained phosphate-releasing injectable purine-crosslinked chitosan-hydroxyapatite constructs
Source: Sci Rep. 2020 Jul 14;10:11603. doi: 10.1038/s41598-020-67886-7 (PMC7360623; doi:10.1038/s41598-020-67886-7)
Supplement: Supplementary file 1 — Supplementary file1 (DOCX 180 kb) [file 41598_2020_67886_MOESM1_ESM.docx]

***In Vitro* and *In Vivo* Investigation of Osteogenic Properties of Self-Contained Phosphate-Releasing Injectable Purine-Crosslinked Chitosan-Hydroxyapatite Constructs**

*Kaushar Jahan^1^, Garthiga Manickam^1^, Maryam Tabrizian^1,2*^ and Monzur Murshed^1,3,4*^*

^1^Faculty of Dentistry, McGill University, Montreal, Quebec, Canada

^2^Department of Biological and Biomedical Engineering, McGill University, Montreal, Quebec, Canada

^3^Department of Medicine, McGill University, Montreal, Quebec, Canada

^4^Shriners Hospital for Children, McGill University, Montreal, Quebec, Canada

**Supplemental Information**

**
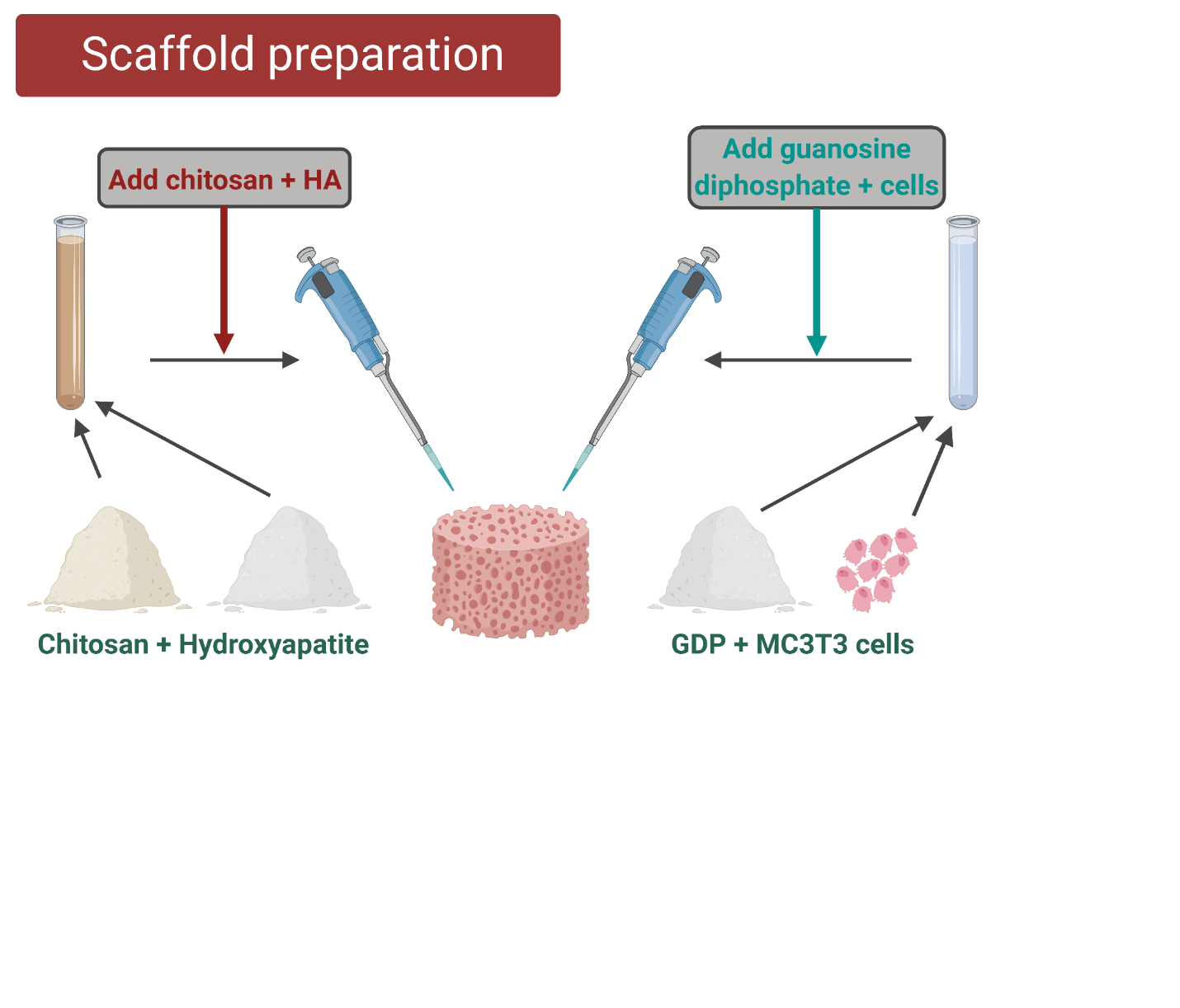
**

**Figure S1.** Preparation of scaffolds

**Table S1.** Cryosection sample preparation

| Solution | Time | Repetition |
| --- | --- | --- |
| PBS | 5 min | 3X |
| 4% formaldehyde | 20 min |  |
| PBS | 5 min | 3X |
| 5% sucrose | overnight |  |
| 20% sucrose | 3 hours |  |
| 50/50 20% sucrose/OCT compound (embedding medium) | 3 hours |  |
